# Peptide-Guided Nanoparticle Drug Delivery for Cardiomyocytes

**DOI:** 10.3390/biology13010047

**Published:** 2024-01-16

**Authors:** Dong Li, Austin Taylor, Haiwang Shi, Fang Zhou, Pengsheng Li, Jyotsna Joshi, Wuqiang Zhu, Shu Wang

**Affiliations:** 1 Department of Cardiovascular Diseases, Physiology and Biomedical Engineering, Center for Regenerative Biotherapeutics, Mayo Clinic Arizona, Scottsdale, AZ 85259, USA; 2 College of Health Solutions, Arizona State University, Phoenix, AZ 85004, USA

**Keywords:** NP, peptide, heart, cardiomyocyte, targeting, drug, delivery

## Abstract

**Simple Summary:**

Heart disease is the leading cause of death worldwide. There is a need to develop a drug delivery system that can specifically target injured hearts and deliver therapeutic agents. Nanoparticles are a promising option for targeted drug delivery in both preclinical and clinical studies. However, the current nanoparticle-based drug delivery system does not have enough specificity in targeting the cardiac tissue. In this study, we have designed cardiac targeting peptides that focus on the molecules present in the cardiomyocyte membrane. Data from cell and animal studies have shown that when nanoparticles are conjugated with these peptides, their binding affinity to cardiomyocytes significantly improves. Therefore, we are developing a targeting system that can be utilized to deliver therapeutic compounds specifically to cardiomyocytes for the treatment of heart diseases.

**Abstract:**

Background: Nanoparticles (NPs) have been extensively utilized as a drug delivery system to control the release of therapeutic agents to treat cardiac injuries. However, despite the advantages of utilizing NP-based drug delivery for treating heart diseases, the current delivery system lacks specificity in targeting the cardiac tissue, thus limiting its application. Methods: We created three linear peptides, each consisting of 16–24 amino acids. These peptides were conjugated on the surface of NPs, resulting in the formation of cardiac targeting peptide (CTP)-NPs (designated as CTP-NP1, CTP-NP2, and CTP-NP3). To assess their effectiveness, we compared the binding efficiency of these three CTP-NPs to human and mouse cardiomyocytes. Additionally, we determined their distribution 24 h after injecting the CTP-NPs intravenously into adult C57BL/6J mice. Results: When compared to control NPs without CTP (Con-NPs), all three CTP-NPs exhibited significantly increased binding affinity to both human and mouse cardiomyocytes in vitro and enhanced retention in mouse hearts in vivo. A thorough assessment of the heart sections demonstrated that the binding specificity of CTP-NP3 to cardiomyocytes in vivo was significantly greater than that of Con-NPs. None of the three CTP-NPs were proven to cause cardiomyocyte apoptosis. Conclusions: Biocompatible and safe CTP-NP3 can target the heart via binding to cardiomyocytes. This approach of targeting specific molecules-coated NPs may help in delivering therapeutic compounds to cardiomyocytes for the treatment of heart diseases with high efficacy and low toxicity to other tissues.

## 1. Introduction

Cardiovascular disease is one of the main causes of death globally [1,2]. Cardiomyocytes in mammalian hearts lose their ability to regenerate soon after birth. When the heart suffers from injuries, it often leads to the formation of scar tissue and decreased heart function. While some medications can help slow down the progression of heart failure, they can also come with undesirable side effects when administered systemically [3]. Therefore, there is an urgent need to develop targeted systems that can deliver therapeutic agents specifically to the heart. This would improve their effectiveness and minimize any unintended effects on other parts of the body.

Nanoparticles (NPs) are particles with a diameter smaller than 100 nm. They can pass through biological barriers, such as capillaries and cell membranes, and deliver cargo to intracellular compartments [4]. Many therapeutic drugs face challenges such as low solubility, lack of stability, high metabolism and elimination rates, short circulation time, lack of target specificity, and potential side effects and toxicity. Nanotechnology can solve many of these issues [5,6]. NPs have been widely used as sustained-release delivery systems for therapeutic agents in the treatment of heart diseases [7]. Our previous research has shown that intramyocardially administering therapeutic agents encapsulated in poly lactic-co-glycolic acid (PLGA) NPs confers cardioprotection via the sustained release of agents to the heart in post-myocardial infarction mice [8]. However, the direct intramyocardial injection of PLGA NPs to the heart is not a convenient or safe administration approach. Intracoronary infusion, pericardial injection, epicardial delivery, intramyocardial injection, and implantable device-based delivery approaches have been used for delivering drugs specifically to the heart [9]. While these approaches have their advantages, such as increasing the local concentrations of therapeutic agents in injured hearts, they often require invasive procedures. Up to now, systemic administration has been the most convenient drug delivery approach to damaged or dysfunctional hearts. For the efficient delivery of therapeutic molecules (such as proteins, peptides, or chemicals) to the heart by intravenous injection, delivery vehicles need to target cardiac cells. There is a critical need to develop NPs that specifically target the heart after systemic administration.

Functionalizing lipid NPs’ surfaces with specific peptides establishes a platform to enhance their advantageous attributes for precision targeting [10,11]. Diminutive molecular ligands, such as short peptides, possess the capacity to covalently affix to lipid surfaces. Previous investigations have documented that short peptides could facilitate the accumulation of drug-loaded lipid NPs at precise sites [12,13]. For example, one previous study employed cardiac targeting peptides (CTPs) for the delivery of siRNA, resulting in a delivery efficiency exceeding two-fold and the noteworthy mitigation of inflammatory manifestations in myocarditis compared to normal small vesicles [14].

Using phage display technology, it was previously found that a long peptide (WLSEAGPVVTVRALRGTGSW) exhibited a high binding affinity of binding to tenascin-X (TNX) at the extracellular matrix of the myocardium [15]. In this study, we modified the peptide into GWLSEAGPVVTVRALRGTGSWGGC for easy conjugation. Since the peptide is long and might be hard to incorporate on the NP surface, we further revised it and developed two short peptides (GVTVRALRGTGSWGGC and GWLSEAGPVVTVRALRGTGGGC) for targeting cardiomyocytes. In addition, we developed biocompatible and biodegradable lipid-NPs using soy phosphatidylcholine, vitamin E, and Kolliphor^®^ HS 15; we incorporated the CTP on the surface of NPs and determined the cardiomyocyte target specificity of three CTP-coated NPs (designated as CTP-NP1, CTP-NP2, and CTP-NP3) both in vitro and in vivo. 

## 2. Materials and Methods

### 2.1. Chemicals and Reagents

Soy phosphatidylcholine (PC) and 1,2-dioleoyl-sn-glycero-3-phosphoethanolamine-N-(lissamine rhodamine B sulfonyl) were purchased from Avanti Polar Lipids Inc. (Cat #1069-79-0 and 810103, Alabaster, AL, USA). Kolliphor^®^ HS 15 and α-tocopherol acetate (α-TA) were purchased from Sigma-Aldrich, Inc. (Cat #42996 and T3001, St. Louis, MO, USA). The three CTPs (GWLSEAGPVVTVRALRGTGSWGGC, GVTVRALRGTGSWGGC, and GWLSEAGPVVTVRALRGTGGGC, respectively) were synthesized by GenScript USA Inc. (Piscataway, NJ, USA). DSPE-PEG2K-maleimide and DSPE-PEG2K (SUNBRIGHT) were purchased from NOF Inc. (Cat #DSPE-020MA and DSPE-020CN, Tokyo, Japan). 1,1′-Dioctadecyl-3,3,3′,3′-Tetramethylindotricarbocyanine Iodide (DiR) was purchased from Thermo Fisher Scientific Inc. (Cat #D12731, San Jose, CA, USA).

### 2.2. Preparation and Characteristics of NPs

Non-targeted lipid NPs (Con-NPs) were prepared using soy PC, α-TA, Kolliphor^®^ HS 15, and 1,2-distearoyl-sn-glycero-3-phosphoethanolamine-N-[(polyethylene glycol)-2000] (DSPE-PEG2k). To render cardiac-targeting capability, we incorporated three linear CTPs (CTP1: GWLSEAGPVVTVRALRGTGSWGGC; CTP2: GVTVRALRGTGSWGGC; and CTP3: GWLSEAGPVVTVRALRGTGGGC) on the NP surface using a maleimide conjugation reaction [16]. CTP-NP1 carries CTP1, CTP-NP2 carries CTP2, and CTP-NP3 carries CTP3. DSPE and PC anchor onto the NP membrane by burying their two fatty acid tails inside the NP core that consists of α-TA, and the PEG2k and conjugated PEK2k-peptide sections protrude towards the outside aqueous environment. The PEG2k helps maintain the integrity and stability of NPs by protecting them from degradation by enzymes and prolonging the circulation of NPs by stabilizing them against opsonization [17,18,19,20]. These peptides can enhance the NP target specificity to cardiac tissues by their highest binding affinity to cardiomyocytes in the heart. 

To prepare for DSPE-PEG2K-peptide conjugates, DSPE-PEG2KPEG2k-maleimide, and three targeting peptides were dissolved at an equal molar ratio in deionized water. The reaction mixture was gently stirred with a magnetic stirrer at room temperature for 24 h. After the reaction, DSPE-PEG2K-maleimide, the targeting peptide, and the DSPE-PEG2k-peptide conjugates (DSPE-PEG2K-CTPs) were characterized by a Bruker Microflex LRF MALDI instrument. To make CTP-NPs, a mixture composed of 7 mg of soy phosphatidylcholine, 22 mg of Kolliphor^®^ HS 15, 22 mg of α-TA, and DSPE-PEG2K (replacing 5 mol% of PC) was dissolved in ethanol. After mixing, ethanol was removed using a nitrogen evaporator. The non-targeted NP lipid mixture was then suspended in warm deionized water and homogenized and sonicated to obtain control NPs without the cardiac targeting peptide (Con-NPs). CTP-NP1, CTP-NP2, and CTP-NP3 were prepared by replacing DSPE-PEG2K with an equal molar mass of DSPE-PEG2K-CTP1, DSPE-PEG2K-CTP2, and DSPE-PEG2K-CTP3, respectively. For the in vitro binding experiment, the fluorescence dye rhodamine was encapsulated into NPs to make rhodamine-labeled NPs. For the in vivo imaging experiments, near-infrared fluorescent DiR dye was encapsulated into NPs to make DiR-labeled NPs. Free (non-encapsulated) dye, peptide, or other compounds were removed by spinning NPs down through a 100kDa Amicon Ultra centrifugal filter (MilliporeSigma Inc., Billerico, MA, USA).

### 2.3. Characteristics of NPs

The particle size, polydispersity indexes (PDIs) and the zeta potential were measured using a Zetasizer Pro (Malvern Panalytical Inc., Malvern, UK). Free (non-encapsulated) dye was separated from nanoencapsulated dye using an ultrafiltration method (Millipore Amicon Ultra-15). Total and free dye concentrations were measured by Cytation 5 (Agilent Inc., Santa Clara, CA, USA). The encapsulation efficiency of dyes in the NPs was calculated using the following equation: Encapsulation efficiency = (Concentration of total dye−Concentration of free dye)/Concentration of total dye × 100%.

### 2.4. Animal Ethics

The animal work was approved by the Institutional Animal Care and Use Committee (IACUC) of Arizona State University (protocol 21-1824R) and Mayo Clinic Arizona (protocol A00007087-23). C57BL/6J mice (8–12 weeks, ~18–28g) obtained from Jackson Laboratory and bred in-house were housed at 22–24 °C and 45% relative humidity, with a daily 12 h light/dark cycle, and had free access to water and a chow diet. Adult male and female mice were placed in the same cage for mating. The neonatal mice (3–5 days after birth) were used for isolating cardiomyocytes.

### 2.5. Evaluation of Tenascin-X Expression in Mouse Organs and Human Cardiomyocytes 

Since the peptides were designed to bind to TNX at the cardiomyocyte membrane, we assessed the expression of TNX in different organs of the mice using Western blotting. We isolated fresh tissues from the heart, liver, kidney, intestine, colon, and uterus of mice. Whole-cell lysates were generated from these tissues as described before [21]. The samples were loaded to 10% SDS-PAGE gel and then transferred to a PVDF membrane. Ponceau S stain was performed to monitor equal protein loading between the samples. Membranes were then blocked in 5% skimmed milk in PBS for 1 h at room temperature and further incubated with primary antibody of TNX (1:3000, Car #XE3591705, Thermo Fisher Inc., Waltham, MA, USA) overnight while shaking at 4 °C. The next day, the membrane was washed three times with phosphate-buffered saline with 0.1% Tween 20 (PBST) and incubated with anti-rabbit horseradish peroxidase-conjugated secondary antibody for 2 h at room temperature (1:10,000, Cat #31460, Thermo Fisher Inc., Waltham, MA, USA). Proteins were visualized using enhanced chemiluminescence reagents and exposed with a ChemiDoc Touch Imaging System (Bio-rad Laboratories Inc., Hercules, CA, USA).

We also evaluated the localization of TNX in the mouse hearts and cardiomyocytes via immunostaining. In brief, heart tissues were cryoprotective and sectioned at 10 μm of thickness. The cardiomyocytes were grown in 2-chamber slides using the protocols described before [22]. The tissue sections and cardiomyocytes were fixed and immunostained with antibodies against TNX overnight at 4 °C. Sections and cells were washed in PBST three times and then incubated with the secondary antibody for one hour at room temperature. Excess antibody was removed by three washes in PBST, and the nuclei were stained with DAPI. Cells were visualized by an Olympus IX80 fluorescent microscope (Olympus Inc., Bartlett, TN, USA).

### 2.6. Evaluation of CTP-NP Binding Affinity to Human-Induced Pluripotent Stem Cell-Derived Cardiomyocytes (hiPSC-CMs) In Vitro 

hiPSCs were purchased from WiCell Research Institute Inc. (Cat #DF-19-9-7T, Madison, WI, USA). The cells were cultured in mTeSR Plus medium (Cat #100-0276, Stem Cell Technologies, Vancouver, Canada) in 6-well plates pre-coated with Matrigel (Cat #354230, Corning Inc., New York, NY, USA) at 37 °C under a humidified atmosphere with 5% CO_2_ as we described before [22]. Cardiomyocyte differentiation was initiated when the cells reached 70–80% confluency. On the first day of differentiation, cells were treated with 6 μM CHIR99021 (Cat #2520691, Biogems Inc., Westlake Village, CA, USA) in RPMI 1640 medium (Cat #10-040-CV, Corning Inc., New York, NY, USA) with 2% of B27 minus insulin (Cat #A18956-01, Gibco Inc., Billings, MT, USA) with a total volume of 3 mL. On day 2, an additional 2 mL of RPMI 1640 with B27 minus insulin was added, and subsequently, 1 mL of RPMI 1640 with B27 minus insulin was added on day 3 of the differentiation. On the next day (day 4), the old media were removed, and the cells were washed with PBS. Then, the cells were treated with 3 mL of 3 μM IWR1 (Cat #1128234, Biogems Inc., Westlake Village, CA, USA) in RPMI 1640 with B27 minus insulin medium. On day 6, the cell media were changed to RPMI 1640 with B27 minus insulin. On day 8, the media were replaced. On day 10, the cells were treated with RPMI 1640 media with 2% B27. Differentiated cardiomyocytes were purified using no-glucose RPMI 1640 media supplemented with 2% B27 and a 4 mM sodium DL-lactate solution (Cat #L7900, MilliporeSigma Inc., St. Louis, MO, USA) for 72 h. Based on our previous experiences, the purity of hiPSC-CMs using this protocol reaches more than 95% [23]. 

For the CTP-NP binding study, the hiPSC-CM cultures at day 28 after the initiation of cardiomyocyte differentiation were utilized. In brief, cells were incubated with NPs at 37 °C, 5% CO_2_ for two hours. Four groups were included. Cells in the Con-NP group received treatment of NPs conjugated with rhodamine (NP^Rhod^), and cells in the remaining three CTP-NP groups received treatment of CTP-NPs conjugated with rhodamine (CTP-NP^Rhod^). The cells were then harvested and fixed with cold acetone and methanol (1:1). Nuclei were identified with DAPI staining. After washing, the samples were mounted with Anti-Fade Fluorescence Mounting Medium (Cat #ab104135, Abcam Inc., Waltham, MA, USA), and the uptake of NP^Rhod^ or CTP-NP^Rhod^ was assessed by fluorescence microscopy. We have previously reported that more than 75% of hiPSC-CMs were mononucleated [23]. Therefore, the rhodamine-positive nuclei were quantified as positive cells in each high-power field, and this number was normalized to the total number of nuclei. Data are presented as a percentage.

### 2.7. Evaluation of CTP-NP Binding Specificity to Mouse Primary Cardiac Cells In Vitro

The hearts were isolated from neonatal mice (C57BL/6J) 3–5 days after birth. Left ventricular cardiomyocytes were isolated as we reported before [24]. In brief, the left ventricle was isolated and rinsed with ice-cold Hanks’ balanced salt solution (HBSS). The ventricular tissues were minced into small pieces (approximately 1 mm^3^) and soaked in 3 mL of 0.05% trypsin-EDTA solution (Cat #BE02-007E, Lonza Inc., Walkersville, MD, USA) for 20 min. The supernatant was discarded, and the pellets were incubated with 10 mL of 0.05% trypsin-EDTA solution in a 37 °C water bath for 5–10 min. The supernatant was collected, and 10 mL of complete DMEM/F-12 culture medium containing 20% fetal bovine serum was added. The digestion step was repeated five times until the heart tissues were digested completely. The supernatant was pooled together and filtered with 100 µm cell strainers. The supernatant was then centrifuged at 300× *g* for 10 min at 4 °C. The cell pellet was resuspended in 3 mL of 1 × red blood cell lysate and incubated at 37 °C for 2 min. After being centrifuged at 300× *g* for 10 min, the cell pellet was resuspended and cultured with complete DMEM/F-12 culture medium containing 10% fetal bovine serum and 10% Penicillin–Streptomycin Solution at 37 °C and 5% CO_2_ in an incubator for 12–16 h.

We used freshly isolated mouse cardiac cells (including cardiomyocytes, endothelial cells, smooth muscle cells, and fibroblasts) to study the efficiency and specificity of small peptide-guided, NP-mediated drug delivery to cardiomyocytes. The primary cardiac cell cultures were incubated with NPs at 37 °C, 5% CO_2_ for two hours. Four groups were included. Cells in the Con-NP group received treatment of NP^Rhod^, and cells in the remaining three CTP-NP groups received treatment of CTP-NP^Rhod^ (final dye concentrations in all four NP groups were 0.2 mg/mL). The cells were then harvested and fixed with cold acetone and methanol (1:1). The cells were immunostained with rabbit monoclonal cardiac troponin T (cTnT) antibody (Cat #ab209813, Abcam Inc., Waltham, MA, USA) at 4 °C overnight. Then, the secondary antibody anti-rabbit IgG was incubated with samples for an additional 1 h at room temperature. Nuclei were identified with DAPI staining. After washing with PBST, the samples were mounted with Anti-Fade Fluorescence Mounting Medium (Cat #ab104135, Abcam Inc., Waltham, MA, USA), and the NP uptake was assessed by fluorescence microscopy. The rhodamine-positive and cTnT-positive cells were quantified and normalized to the total number of rhodamine-positive cells (both cardiomyocytes and nonmyocytes), and the data are presented as a ratio. 

### 2.8. Biodistribution and Cardiac-Target Specificity of NPs in Mice

A total of 20 mice (C57BL/6, Jackson Lab; 8–12 week, ~18–28g) were used in this study. We encapsulated DiR, a near-infrared dye (λ of excitation is 730 nm; λ of emission is 790 nm), into Con-NPs (Con-NP^DiR^) and CTP-NPs (CTP-NP^DiR^). CTP-NP^DiR^ and Con-NP^DiR^ were suspended in 50 μL of normal saline for subsequent in vivo use. Mice were then restrained, and CTP-NPs or Con-NPs containing the same amounts of DiR were intravenously injected into mice via tail veins using a Hamilton syringe with a sterile 30.5-gauge needle. Then, 2 h and 24 h after the injections, mice were separately imaged using an in vivo imaging system (IVIS) with a near-infrared dye filter. After the second time of scanning, mice were euthanized by an overdose of isoflurane inhalation and subsequent cervical dislocation. After heart perfusion from the left ventricle with 1 × PBS, the hearts, lungs, livers, spleens, and kidneys were harvested. Then, the DiR fluorescence intensity of these organs was acquired using the IVIS system.

After scanning, the mouse hearts were embedded into OCT compounds and processed for cryosectioning at 10 µm thickness. The cardiomyocytes in the heart sections were labeled via anti-cTnT immunostaining. To evaluate the binding specificity of CTP-NPs, both DiR- and cTnT-positive cells were quantified and normalized to the total number of cardiomyocyte nuclei; the data were presented as a ratio. The DiR signal was evaluated for every five slides using the Olympus IX80 fluorescent microscopy (Evident Scientific, Inc., Waltham, MA, USA) with a Cy7 (or far red) filter. 

### 2.9. Evaluation of CTP-NP Toxicity to Heart Tissue via TUNEL Staining

Cardiomyocyte apoptosis was evaluated on mouse heart tissue sections by using the ApopTag Apoptosis Detection kit as directed by the manufacturer’s instructions (Cat #S7100, Chemicon International, Billerica, MA, USA). In brief, heart sections were fixed with 4% paraformaldehyde for 10 min at room temperature, washed in PBS, and incubated with 75 µL of equilibration buffer for 5 min at room temperature. The cells were then incubated with 55 µL of working-strength TdT enzyme for 1 h at 37 °C, washed with working-strength stop buffer for 10 min, and then by three changes of PBS. The samples were incubated with anti-digoxigenin conjugate for 30 min at room temperature and washed in PBS. Slides were mounted with medium containing DAPI (Cat #H-1200, Vector Laboratories Inc., Newark, CA, USA). Cardiomyocytes were labeled using anti-cTnT immunostaining. TUNEL-positive/cTnT-positive cells were quantified and normalized to the total number of cardiomyocyte nuclei. Data were presented as a ratio.

### 2.10. Statistical Analysis

All data were presented as Mean ± SEM. Student’s *t*-test was used for comparison between two groups, and one-way ANOVA with a Holm–Sidak post hoc test was used for comparison between multiple groups. *p* < 0.05 was considered significant. Data from at least three repeats were collected for each of the in vitro experiments.

## 3. Results

### 3.1. Design of Cardiac Targeting Peptides

**Figure 1:**
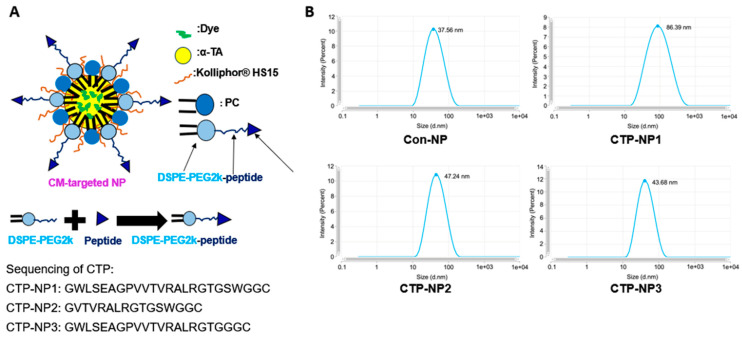
CTP-NP structure and size. (**A**) Illustration of the CTP-NP design. (**B**) The sizes of non-targeted NPs and targeted NPs were evaluated using a transmission electron microscope.

Using phage display technology, McGuire et al. previously found that a long peptide (WLSEAGPVVTVRALRGTGSW) exhibited a high binding affinity to primary cardiomyocytes. This phage peptide contains a 12-amino-acid segment with a sequence identical to a peptide in TNX, which is a member of the tenascin family and is expressed in the extracellular matrix of mouse myocardia [15]. However, since the peptide is long, it is hard to incorporate it on the NP surface. Here, we modified the peptide to make GWLSEAGPVVTVRALRGTGSWGGC and made two shorter peptides (GVTVRALRGTGSWGGC and WLSEAGPVVTVRALRGTGGGC, respectively) on the surface of lipid NPs. PEG2k-DSPE and PC anchor onto the NP membrane by burying their two fatty acid tails inside the NP core (Figure 1A). At the same time, the PEG2k and conjugated peptide of PEK5k sections protrude towards the outside aqueous environment. The cardiac targeting peptide, conjugated on the top of PEG2k, also protrudes towards the external environment. CTP-NP1 carries the targeting peptide GWLSEAGPVVTVRALRGTGSWGGC, CTP-NP2 carries the targeting peptide GVTVRALRGTGSWGGC, and CTP-NP3 carries the targeting peptide GWLSEAGPVVTVRALRGTGGGC. The non-targeted Con-NPs were NPs without CTP conjugation. The Con-NPs (non-targeted) exhibited an average diameter of 37.56 nm, CTP-NP2 and CTP-NP3 were around 47.24 and 43.68 nm, respectively. Since peptide 1 is long, the average size of CTP-NP1 was around 86.39 nm (Figure 1B). The dye encapsulation efficiency was more than 95% in all NPs. 

**Figure 2:**
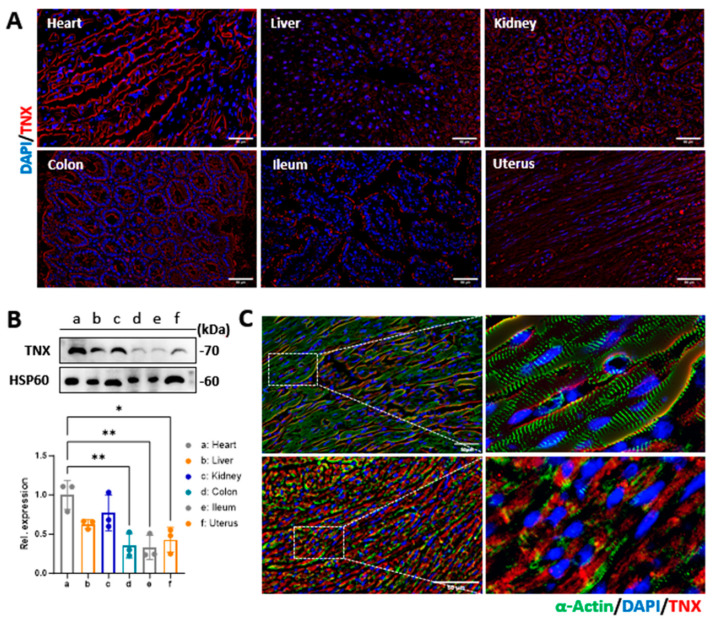
Expression of TNX in mouse organs and cellular localization in the mouse myocardial tissue. (**A**) Expression of TNX in organs (heart, liver, kidney, colon, ileum, and uterus) was evaluated by immunostaining and Western blot. Bar = 50 μm. (**B**) Expression of TNX in organs (heart, liver, kidney, colon, ileum, and uterus) was evaluated by Western blot. Data were presented as mean ± SEM. n = 3 in each group, one–way ANOVA with Holm–Sidak post hoc test, * *p* < 0.05, ** *p* < 0.01, (**C**) Cellular localization of TNX in the mouse myocardium was determined via immunostaining. Bar = 50 μm.

We assessed the expression of TNX in different organs of the mice since the peptides were designed to bind to TNX at the cardiomyocyte membrane. Data from the anti-TNX immunostaining of mouse organs (Figure 2A) and Western blot analysis (Figure 2B) showed that TNX is highly expressed in the heart, with some expression in the liver, kidney, colon, intestine, and uterus. We also studied the cellular localization of TNX in the myocardium via anti-TNX immunostaining in the mouse myocardial tissue. Our data showed that the TNX is highly enriched in the plasma membrane of mouse cardiomyocytes (Figure 2C), which is different from previous reports that TNX is a molecule in the extracellular matrix of the myocardium [15].

### 3.2. Conjugation with CTP Enhances the Targeting Efficiency of NPs to hiPSC-CMs and Primary Mouse Cardiomyocytes

**Figure 3:**
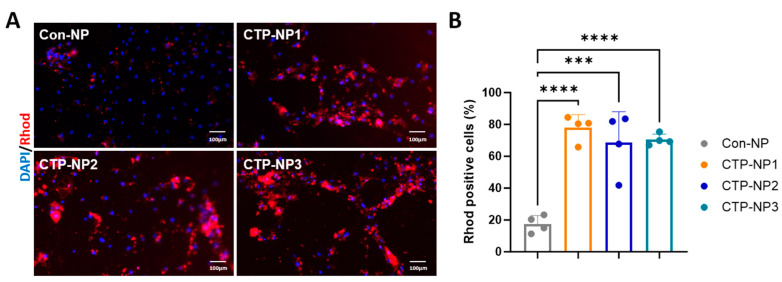
Evaluation of CTP-NP binding affinity to hiPSC-CMs in vitro. (**A**) The binding affinity of CTP-NP^Rhod^ to hiPSC-CMs was evaluated by immunofluorescence. NPs were conjugated to rhodamine. Cardiomyocytes were marked by immunostaining with antibodies against cTnT. Nuclei were counterstained with DAPI. Bar = 100 μm. (**B**) The number of rhodamine and cTnT double-positive cells were counted and normalized to the total number of cardiomyocyte nuclei, and the data were presented as a percentage. Data were presented as mean ± SEM. n = 4 for each group. Statistical analysis was performed using one-way ANOVA with a Holm–Sidak post hoc test. *** *p* < 0.005, **** *p* < 0.001.

We compared the binding affinity of NPs with or without CTP conjugation to hiPSC-CMs. The Con-NPs and three types of CTP-NPs were conjugated with rhodamine to track the cellular localization of NPs. At 28 days after the initiation of cardiac differentiation, the hiPSC-CMs were treated with Con-NPs and CTP-NP1, CTP-NP2, and CTP-NP3 for two hours. After NP treatment, the cells were fixed and then labeled via immunostaining with DAPI (Figure 3A). We quantified the number of rhodamine-positive cells, and the results were presented as a percentage of total cells. Our data revealed that the binding affinity to hiPSC-CMs of all three CTP-NPs (i.e., CTP-NP1, CTP-NP2, and CTP-NP3) was significantly increased compared to that of Con-NPs (Figure 3B). No significant difference was observed between the CTP-NP groups.

**Figure 4:**
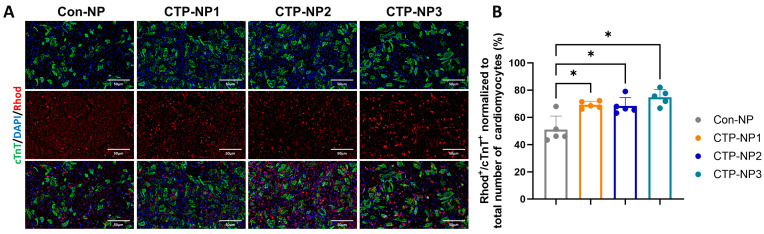
Evaluation of CTP-NP binding specificity to mouse cardiomyocytes in vitro. (**A**) The binding specificity of CTP-NP^Rhod^ to primary mouse cardiac cells (including both myocytes and nonmyocytes) was evaluated by immunofluorescence. NPs were conjugated to rhodamine. Cardiomyocytes were marked by immunostaining with antibodies against cTnT. Nuclei were counterstained with DAPI. Bar = 50 µm. (**B**) The number of rhodamine and cTnT double-positive cells was counted and normalized to the total number of cardiomyocyte nuclei, and the data were presented as a percentage. Data were presented as mean ± SEM. n = 5 for each group. Statistical analysis was performed using one-way ANOVA with a Holm–Sidak post hoc test. * *p* < 0.05.

We also evaluated the binding specificity of CTP-NPs to primary mouse cardiomyocytes in vitro. NPs were conjugated with rhodamine. The primary mouse cardiomyocytes were treated with Con-NPs, CTP-NP1, CTP-NP2, and CTP-NP3 for 2 h. After NP treatment, the cells were fixed. Cardiomyocytes were labeled via immunostaining with antibody against cTnT (Figure 4A). We quantified the number of rhodamine and cTnT double-positive cells and normalized it to the total rhodamine-positive cells, and the results were presented as a percentage. Our data revealed that the binding affinity to primary mouse cardiomyocytes of all three NPs conjugated with CTPs (i.e., CTP-NP1, CTP-NP2, and CTP-NP3) was significantly increased compared to that of Con-NPs, whereas there was no difference between the three groups of CTP-NPs (Figure 4B).

### 3.3. Conjugation with CTP Elevates the Targeting Efficiency of NPs to Heart in Mice

**Figure 5:**
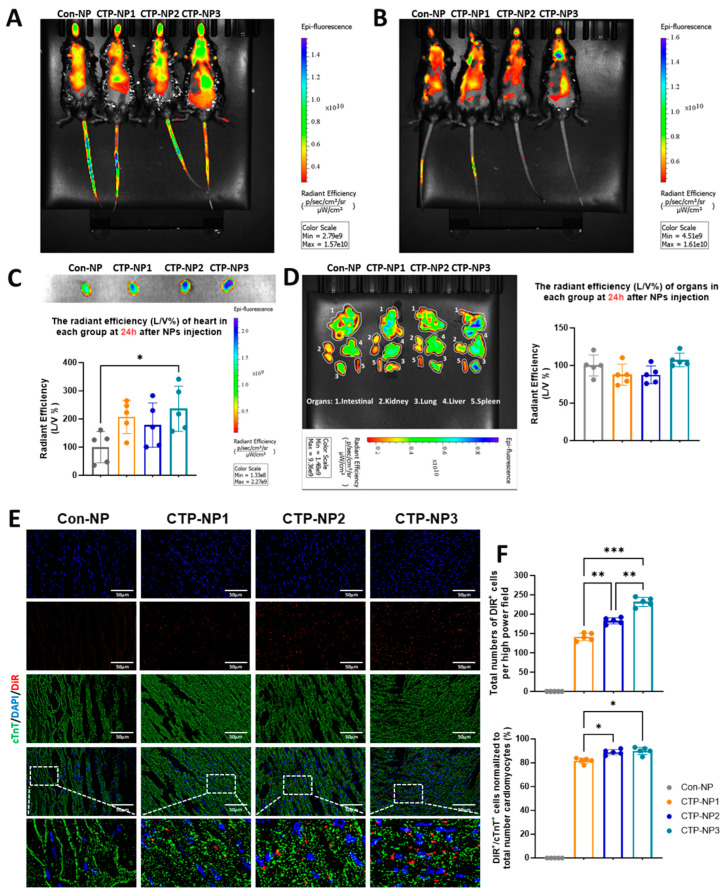
Evaluation of CTP-NP binding to mouse cardiomyocytes in vivo. Three CTP-NPs (CTP-NP1, CTP-NP2, and CTP-NP3) and NPs (Con-NPs) were conjugated with DiR and were subsequently injected into mice via tail vein. (**A**,**B**) Representative images of bioluminescence assay at 2 h and 24 h, respectively, after NP injection. (**C**,**D**) The animals were euthanized, and the organs (heart, intestinal, kidney, lung, liver, and spleen) were isolated 24 h after NP injection, then subjected to bioluminescent assay. Data were presented as mean ± SEM. n = 5 for each group. Statistical analysis was performed using one–way ANOVA with Holm–Sidak post hoc test. * *p* < 0.05. (**E**) Hearts were processed for cryosectioning at 10 µm thickness. The binding specificity of CTP-NP to mouse cardiac cells (including both myocytes and nonmyocytes) was evaluated by immunofluorescence. Cardiomyocytes were marked by immunostaining with antibodies against cTnT. Nuclei were counterstained with DAPI. Bar = 50 µm. (**F**) The number of DiR- and cTnT-positive cells was quantified and normalized to the total number of cardiomyocyte nuclei, and the data were presented as a percentage. Data were presented as mean ± SEM. n = 5 for each group. Statistical analysis was performed using one–way ANOVA with a Holm–Sidak post hoc test. * *p* < 0.05, ** *p* < 0.01, *** *p* < 0.005.

We evaluated the binding specificity of CTP-NPs to mouse cardiomyocytes in vivo via two different approaches. The same amount of DiR dye was encapsulated into Con-NPs and three types of CTP-NPs to determine the biodistribution and target specificity of NPs in vivo. The NPs were intravenously injected into adult mice via the tail vein. The DiR was detected in mice using the IVIS system at hour 2 (Figure 5A) and 24 (Figure 5B) post injection. The results showed that the dye intensity of the CTP-NP groups significantly increased in hearts, with CTP-NP3 having a higher dye intensity than Con-NPs, CTP-NP1, and CTP-NP2 at both hour 2 and 24. The animals were euthanized after the second IVIS scanning (24 h after injection), and organs (heart, intestine, kidney, lung, liver, and spleen) were harvested.

**Figure 6:**
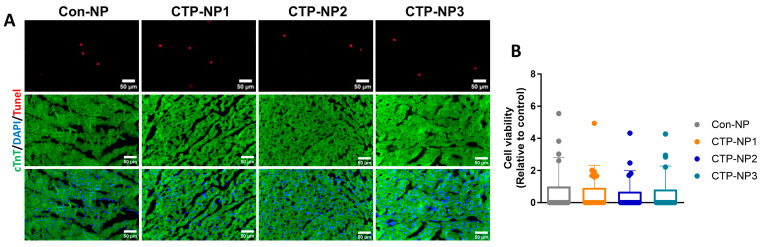
Evaluation of the potential toxicity of CTP-NPs. (**A**) Cardiomyocyte apoptosis was evaluated by TUNEL staining. Nuclei were counterstained with DAPI. Bar = 50 µm. (**B**) The number of TUNEL-positive nuclei was counted and normalized to the total number of DAPI-positive nuclei, and the data were presented as a percentage. Data were presented as mean ± SEM. n = 15 for each group. Statistical analysis was performed using one–way ANOVA with Holm–Sidak post hoc test.

First, the DiR fluorescent intensity in different organs was quantified with IVIS imaging. Our data revealed that 24 h after NP injection, animals receiving the CTP-NP3 injection displayed significantly increased DiR signal intensity in their hearts as compared to Con-NPs (Figure 5C), but no significant difference was observed in other organs (Figure 5D). Second, the DiR signal intensity was evaluated in the heart sections under an epifluorescent microscope. The mouse cardiomyocytes in heart tissue sections were labeled with the immunostaining of anti-cTnT antibody. The DiR and cTnT double-positive cells were normalized to total DiR-positive cells, and the results were presented as a percentage (Figure 5E,F). Our data revealed that all three groups of CTP-NPs showed significantly increased DiR signal intensities in cardiomyocytes. Among the three groups of CTP-NPs, CTP-NP2 and CTP-NP3 displayed higher specificities than CTP-NP1, and there was no difference between CTP-NP2 and CTP-NP3 (Figure 5F). We also evaluated the potential toxicity of CTP-NPs via TUNEL staining with heart cryosections (Figure 6A). Our results showed that none of the three CTP-NPs induced cardiomyocyte apoptosis (Figure 6B). Together, these data suggested that CTP conjugation enhances the targeting specificity of NPs to cardiomyocytes in vivo without inducing cardiac toxicity.

## 4. Discussion

There is increasing interest in utilizing drug delivery systems to improve the efficiency and safety of the delivery of medications and other therapeutic substances to the heart for treating cardiovascular disease. Nanomedicine and nano-delivery systems hold considerable potential in efficiently delivering therapeutic drugs to specific areas within the body. These systems can offer targeted, controlled, and prolonged drug delivery, with increased efficacy and decreased side effects and toxicity within the body [25,26]. In this study, we created a new heart-targeted NP-based delivery system. We used lipid-NPs as a drug carrier and three CTPs on the surface of NPs to guide the heart target and delivery. Our results showed that these CTP-NPs have the ability to deliver therapeutic substances directly to the hearts of living mice through intravenous injection. The information obtained from this study forms the basis for the development of delivery systems that can be used to treat cardiovascular diseases. Importantly, this development enables materials to be injected directly into the bloodstream, resulting in targeted accumulation and retention in the heart.

Targeting strategies can be divided into two main categories, namely passive targeting and active targeting [9]. Passive targeting relies on the physicochemical properties of cells and the surrounding microenvironment in specific situations like leaky blood vessels. On the other hand, the active targeting strategy involves the use of cell-specific ligands that facilitate precise and highly effective binding to receptors or transporters located on the plasma membrane of cardiac cells. It was reported that the modification of the NP surface by conjugating with certain peptides, proteins, or ligands may increase their affinity to the membrane receptors/transporters of specific cells [27]. In the current study, we chose three peptides with sequence identity to TNX, a multi-domain protein that includes 24 fibronectin-motif domains [28]. Different members of the tenascin family are present in various tissues of the body. It was reported that TNX is present in the extracellular matrix of the myocardium [29]. Consistent with these reports, we observed that the expression of TNX in the heart is significantly higher compared to other tissues. However, our data showed that TNX is highly enriched in the plasma membrane of cardiomyocytes. Additionally, we found that all three NPs coupled with CTPs showed a significant increase in binding affinity to cardiomyocytes, both in vitro and in vivo, when compared to the control NP without CTP coupling. Furthermore, we assessed the biodistribution of the NPs in mice 2 and 24 h after injection through the tail vein. Our findings showed higher bioluminescent intensity in these NPs compared to Con-NPs, and importantly, we observed no toxicity to the myocardium. In summary, we developed a targeted delivery system specifically for the heart and demonstrated its potential for treating cardiac diseases.

It was reported that biophysical properties of the nanoparticles and tissue microenvironment may affect the nanoparticle bioavailability [4]. Since the heart is a highly perfused organ, bloodstream effects may increase the delivery of nanoparticles to the heart. Indeed, we observed the increased biodistribution of NPs in the highly perfused organs such as the lungs, liver, kidney, and intestine two hours after intravenous injection to mice. However, we observed an enrichment of NPs in the heart 24 h later. In addition, we confirmed the increased expression of the TNX receptor in the heart through immunostaining and Western blotting analysis. Furthermore, the CTP-NPs showed increased binding to the cardiomyocytes in vitro and in vivo compared to control NPs (with CTP conjugation). Therefore, it is unlikely that the increased nanoparticle signal in the heart is solely due to the effects of the bloodstream or tissue microenvironment.

Lipid NPs have also been widely used as promising approach for delivering therapeutics into an injured heart because they have a similar structure to cell membranes [30]. Zhou et al. conducted an experiment where they loaded lipid NPs with collapsin response mediator protein 2 (CRMP2) small interfering RNA and injected them into a mouse model of chronic myocardial infarction via the tail vein. The treatment resulted in a change in the type of macrophages present in the wound from M1 to M2, a significant decrease in inflammation and fibrosis, and a notable improvement in heart function and survival rate [31]. Cyclosporine lipid NPs were used to prevent the opening of the mitochondrial permeability transition pore. As a result, there was a decrease in necrosis and apoptosis of cardiomyocytes in the early phase of ischemic injury [32]. The direct and effective method of delivering medical treatments directly into the heart is through an injection into the heart muscle. However, there are limitations to the amount of substance that can be injected, and this method is invasive.

Cheng and his colleagues have created a detachable microneedle patch for therapy. The patch is made up of microneedles composed of a gel called elastin-like polypeptide. These microneedles are filled with NPs that contain factors secreted by mesenchymal stromal cells. The base of the microneedles is made of a gel called hyaluronic acid, which can be easily dissolved. Once the base is removed, the tips of the microneedles can be inserted securely into the damaged tissue of the heart. This insertion helps to reduce cell death, restore the volume of the myocardium, and decrease the formation of scar tissue during the process of repairing the heart [33]. Despite the significant progress in this field, the efficiency of delivering lipid NPs inside cells is still relatively low. The continuous concerns regarding safety and the possibility of evoking an immune response due to synthetic lipid components make it necessary to study different compositions of lipid NPs [34]. In the present study, we created a distinct delivery system that accurately directs drugs toward the heart, thereby ensuring effective and targeted drug delivery to the hearts of living animals. This system consistently maintains elevated levels of drug distribution within the heart. This breakthrough improves the convenience and precision of drug delivery systems concerned with the heart and introduces novel opportunities for treating cardiac injuries.

This study is a proof-of-concept study. One limitation of this study is that we did not test the capability of the CTP-NPs to carry a variety of cargoes. However, we demonstrated the ability of the CTP-NPs to carry fluorescent dyes (such as rhodamine and DiR) across the cellular membranes in intact, functional cardiomyocytes in vitro and in live animals. Future studies are necessary to investigate whether the delivery of cardioprotective drugs by CTP-NPs preserves heart function in animal models of heart injuries. Second, NPs in blood need to cross the endothelial cells of capillary vessels to reach the myocardium. We did not test if conjugation with CTPs affects the adhesion of NPs to vascular endothelial cells or their ability to cross the vascular barrier in the heart. Third, though CTP-NPs did not increase cardiomyocyte apoptosis in our study, additional experiments to assess the safety of CTP-NPs in vivo are warranted.

## 5. Conclusions

In conclusion, the three innovative CTP-NPs we developed, CTP-NP1, CTP-NP2, and CTP-NP3, were made of phospholipids, vitamin E, Kolliphor^®^ HS 15, and DSPE-PEG2k-peptide. Their cardiac target specificity was determined in mice using an IVIS system and in cell culture models. The findings reveal that all three CTP-NPs demonstrate markedly enhanced binding affinity to both human and mouse cardiomyocytes compared to Con-NPs. Notably, CTP-NP3 shows superior binding specificity to the heart via binding to cardiomyocytes, which is a crucial step toward achieving targeted therapy for cardiac injuries. Importantly, none of the CTP-NPs induce cardiomyocyte apoptosis. CTP-NP3, in particular, holds great potential for targeted cardiac therapy, offering high efficacy in delivering therapeutic agents directly to cardiomyocytes while minimizing toxicity to other tissues. This study presents a significant advancement in targeted drug delivery for heart disease treatments and paves the way for more effective and safer treatments for heart diseases.

## Data Availability

All data are kept in the laboratories of Shu Wang and Wuqiang Zhu and can be made available upon reasonable request.
